# Upregulation of the long noncoding RNA UCA1 affects the proliferation, invasion, and survival of hypopharyngeal carcinoma

**DOI:** 10.1186/s12943-017-0635-6

**Published:** 2017-03-21

**Authors:** Ye Qian, Dayu Liu, Shengda Cao, Ye Tao, Dongmin Wei, Wenming Li, Guojun Li, Xinliang Pan, Dapeng Lei

**Affiliations:** 1 Department of Otorhinolaryngology, Qilu Hospital, Shandong University; Key Laboratory of Otolaryngology, NHFPC (Shandong University), 107 West Wenhua Road, Jinan, Shandong 250012 People’s Republic of China; 2 Department of Otolaryngology & Head and Neck Surgery, 2nd Affiliated Hospital of Anhui Medical University, Hefei, China; 3 Department of Head and Neck Surgery, The University of Texas MD Anderson Cancer Center, Houston, TX 77030 USA; 4 Department of Epidemiology, The University of Texas MD Anderson Cancer Center, Houston, TX 77030 USA

**Keywords:** Long noncoding RNAs, UCA1, Hypopharyngeal squamous cell carcinoma, Prognosis

## Abstract

**Background:**

Several long noncoding RNAs (lncRNAs) are involved in oncogenesis.

**Methods and Results:**

Our microarray analysis showed that numerous lncRNAs are dysregulated in hypopharyngeal squamous cell carcinoma (HSCC) tumor tissues as compared with normal tissues. Among those lncRNAs, urothelial carcinoma-associated 1 (UCA1) has been found to have an oncogenic role in HSCC. We confirmed the upregulation of UCA1 in HSCC by assessing its expression levels in a cohort of 53 patient tumors and paired non-tumor samples. In addition, we found that high UCA1 expression was significantly associated with advanced T category, late clinical stage, greater lymphatic invasion, and worse prognosis. Furthermore, in vitro experiments demonstrated that UCA1 functioned as an oncogene by promoting the proliferation and invasion and preventing the apoptosis of HSCC cells.

**Conclusions:**

Taken together, our findings for the first time identify the role of UCA1 as a tumor promoter and a pro-metastatic factor in HSCC, demonstrating that UCA1 is a potential prognostic biomarker and therapeutic target in HSCC.

## Background

Hypopharyngeal squamous cell carcinoma (HSCC), a malignant tumor arising from the mucosa of the upper aerodigestive tract, accounts for 5% of all squamous cell carcinomas in the head and neck [1, 2]. Patients with early-stage HSCC have a 5-year overall survival rate as high as 70% [3]; however, HSCC is seldom diagnosed at its early stages because early-stage HSCC causes no obvious symptoms. Thus, approximately 70–85% of HSCCs are diagnosed at stage III or IV, and the 5-year overall survival rate of patients with stage III or IV HSCC is only 15–45% [4]. Despite recent improvements in available treatment strategies for HSCC, which include primary surgery with pre- or postoperative radiotherapy and primary radiotherapy with chemotherapy, the prognosis of HSCC patients with late-stage disease is still far from satisfactory. Therefore, it is imperative to identify sensitive and specific progression and prognostic biomarkers that facilitate the early diagnosis of HSCC and might serve as potential therapeutic targets in the disease.

Long noncoding RNAs (lncRNAs)—non–protein-coding RNAs longer than 200 nucleotides—may serve as novel biomarkers and therapeutic targets in cancer. Previous studies have shown that lncRNAs are pervasively transcribed and that some lncRNAs can regulate gene expression at transcriptional and posttranscriptional levels by interacting with nucleic acids or proteins [5–7]. In addition, lncRNAs have been widely implicated in diverse biological processes, including cell proliferation, cell differentiation, and chromosome inactivation [8, 9]. Increasing evidence demonstrates that various cancers, including breast cancer, lung cancer, pancreatic cancer, osteosarcoma, hepatocellular carcinoma, and leukemia, have aberrant lncRNA expression [10–13].

One of lncRNAs that show promise as a potential biomarker of HSCC is urothelial carcinoma-associated 1 (UCA1). UCA1 is a well-characterized lncRNA that was initially identified in urinary bladder cancer tissues, where it was found to significantly enhance bladder cancer cells’ tumorigenicity and invasive potential in vitro and in vivo [14]. UCA1, which also functions as a tumor promoter in breast cancer, colorectal cancer, gastric cancer, and esophageal squamous cell carcinoma [15–18], is closely related to the prognosis of a variety of cancers; for example, Han et al. found that high UCA1 expression indicated worse prognosis in colorectal cancer [16]. In a previous microarray analysis, we found that UCA1 was one of mostly overexpressed in HSCC tissue [19]. However, the role of UCA1 in HSCC remains unclear.

In this study, we first validated the expression of several mostly altered lncRNAs in HSCC patients; **we found that UCA1 is one of mostly upregulated (top 3) in HSCC tissues compared with adjacent non-tumor tissues and then determined that its expression is closely linked to HSCC patients’ clinicopathological characteristics and prognosis**. Furthermore, in vitro experiments established that UCA1 has roles in cell apoptosis, proliferation, and invasion in HSCC, suggesting that UCA1 is an important prognostic biomarker and treatment target for HSCC.

## Methods

### Patients and tissue samples

HSCC specimens and corresponding adjacent normal tissues were obtained from 53 HSCC patients who underwent surgery in the Department of Otolaryngology at the Qilu Hospital of Shandong University from March 2011 to December 2011. All HSCC specimens were confirmed by pathological diagnosis, and all patients underwent hypopharyngeal tumor resection plus ipsilateral modified neck dissection. None of the 53 patients received chemoradiotherapy or biotherapy before surgery. All patients received radiotherapy after surgery.

The clinicopathological information were obtained from patients’ history record including patient age, overall survival duration, tumor cell differentiation, T category, clinical disease stage, and lymph node status. The TNM staging system follows the standard of the International Union Against Cancer. Patients returned for follow-up visits once every 3 months until March 2016; 4 patients were lost to follow up. Before undergoing surgery, all patients provided their written informed consent for their tissues to be used in the study. The study protocol was approved by the Ethics Committee of the Qilu Hospital of Shandong University.

### Cell cultures

The only available HSCC cell line, FaDu, was purchased from the Shanghai Institute of Biology and maintained in Dulbecco’s modified Eagle’s medium supplemented with 10% fetal bovine serum (FBS; Gibco, Grand Island, NY, USA) in a humidified atmosphere containing 5% CO_2_ at 37°C.

### RNA extraction and quantitative real-time polymerase chain reaction

To extract total RNA from human tissue samples, we used Trizol reagent (Takara, Otsu, Japan) according to the manufacturer’s instructions. We used a NanoDrop ND-1000 spectrophotometer (Thermo Scientific, Wilmington, DE, USA) to measure RNA concentration and purity. We subjected 2 μg of RNA from each tissue sample to cDNA synthesis using a reverse transcription kit (Takara). Quantitative real-time polymerase chain reaction (qRT-PCR) was performed with SYBR Green Real-Time PCR Master Mix (Toyobo, Osaka, Japan) and the LightCycler 480 II System (Roche, Basel, Switzerland). UCA1 and β-actin were amplified in triplicate using an annealing temperature of 60°C. Signals were quantified using the ΔΔCt method and normalized to β-actin levels.

### Construction of lentiviral vectors

**Fig. 1:**
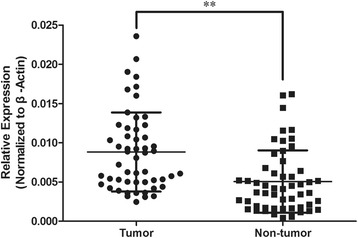
UCA1 expression levels in HSCC tissues and paired adjacent normal tissues. qRT-PCR was used to assess the relative expression of UCA1 in two groups of samples, and β-actin was used as a normalization control. *n* = 53; **P < 0.01

DNA oligonucleotides to produce plasmid-based small hairpin RNA (shRNA) were cloned into the pLeno–green fluorescent protein (GFP) vector. The shRNA target sequences for UCA1 were GGACAACAGTACACGCATA (sh-1) and TTAATCCAGGAGACAAAGA (sh-2); a scrambled sequence, which does not share obvious homology with human gene sequences, was used as a negative control. The pLeno-GFP vector and packing vectors (pRsv-REV, pMDIg-pRRE, and PMD2G) were cotransfected into 293T cells using Lipofectamine 2000 according to the manufacturer’s instructions. After 48 h, the supernatant was collected, concentrated by centrifugation, and filtered through 0.45-μm cellulose acetate filters. The titer was measured by counting the GFP-positive cells under a fluorescence microscope (Olympus, Tokyo, Japan). FaDu cells in 6-well plates were infected with the constructed lentiviruses at a multiplicity of infection of 60. The infection efficiency was determined by counting the GFP-positive cells under a fluorescence microscope 96 h after infection. The efficiency of UCA1 silencing was subsequently quantified by qRT-PCR (Fig. 1).

### Apoptosis analysis

The Annexin V–allophycocyanin apoptosis detection kit (eBioscience, San Diego, CA, USA) was used to identify apoptotic cells. Briefly, FaDu cells were seeded in 6-well plates following 5 days of lentiviral infection at a multiplicity of infection of 60. After being trypsinized and washed twice with phosphate-buffered saline (PBS), the FaDu cells were stained with Annexin V–fluorescein isothiocyanate and propidium iodide (PI) successively in the staining buffer at 4°C in the dark and then detected by flow cytometry with the FACSCalibur system (BD Biosciences, San Diego, CA, USA).

### Cell cycle analysis

FaDu cells were seeded in 60-mm dishes and infected with the lentivirus for 24 h. Cells were harvested and washed twice with cold PBS and then fixed with ice-cold 70% ethanol for 1 h at 4°C. After centrifugation at 1500 r/min for 5 min, the cells were washed twice with PBS and resuspended with 0.5 ml of PBS containing PI (50 μg/ml) and Rnase (100 μg/ml). The cells were then stained at room temperature in the dark for 15 min, and the cell cycle distribution was assessed by flow cytometry and analyzed using ModiFit 3.0 software (Becton Dickinson).

### Colony formation assay

We used a colony formation assay to assess the effect of UCA1 knockdown on FaDu cell colony formation. Briefly, stably transfected FaDu cells were sufficiently trypsinized and suspended in DMEM supplemented with 10% FBS. The cells were then seeded in 6-well plates in triplicate (800 cells/well) and maintained in a humidified atmosphere containing 5% CO_2_ at 37°C. The medium was changed every 3 days. After culture for 14 days, cell colonies were washed with PBS, fixed with 4% paraformaldehyde for 30–60 min, and then stained with Giemsa (ECM550 Chemicon, CA, USA) for 20 min. Only colonies containing more than 50 cells were counted.

### Transwell assays

To assess the invasiveness of FaDu cells, we used transwell chambers (Corning, Tewksbury, MA, USA) coated with Matrigel (BD Biosciences). Fully trypsinized, transfected cells (1 × 10^5^ cells in 100 μl of serum-free DMEM) were plated into the upper chamber. DMEM (500 μl) supplemented with 20% FBS was added to the lower chamber. After incubation in a humidified atmosphere containing 5% CO_2_ at 37°C for 72 h, FaDu cells that had invaded the lower chamber were fixed with methanol and stained with Giemsa. We used an inverted microscope (magnification 200×) to count the invading cells manually.

### Statistical analysis

All experiments were repeated at least 3 times. Data were presented as the mean ± the standard deviation and analyzed with the SPSS software program (version 20.0). *P* values < 0.05 were considered statistically significant. The relationship between clinicopathological characteristics and UCA1 expression was analyzed using the chi square test or Fisher exact test. The Student *t* test was used to assess differences between two experiment groups. We used the Kaplan-Meier method to create survival curves for patients with high or low UCA1 expression and used a log-rank test to compare overall survival rates.

## Results

### UCA1 is overexpressed in HSCC tumor tissues

To confirm that UCA1 is upregulated in HSCC tumor tissues, we performed qRT-PCR to measure the relative expression levels of UCA1 in 53 pairs of HSCC tumor tissues and their corresponding adjacent non-tumor mucosae. The relative expression level of UCA1 in the HSCC tissues (0.0088 ± 0.0050) was significantly higher than that in the paired adjacent normal tissues (0.0051 ± 0.0040, *P* < 0.05; Fig. 1), which suggests that UCA1 is involved in the pathogenesis of HSCC.

### Association between UCA1 expression and clinicopathological characteristics and survival

**Fig. 2:**
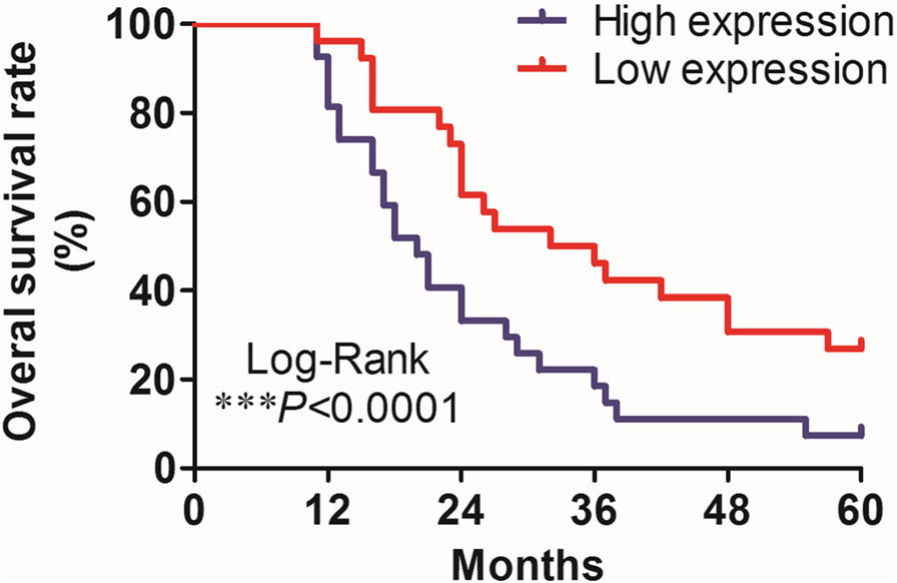
Kaplan–Meier analysis demonstrated that patients with high UCA1 expression had a significantly worse prognosis than those with low UCA1 expression did. ***P < 0.0001, log-rank test

To further investigate the relationship between the UCA1 expression level and clinicopathological characteristics, we divided the 53 patients into high– and the low–UCA1 expression groups according to the patients’ overall median UCA1 expression level. As shown in Table 1, high UCA1 expression levels were significantly correlated with advanced T category (*P* = 0.034), late clinical stage (*P* = 0.021), and worse lymph node metastasis (*P* = 0.02). However, we found no significant correlation between UCA1 expression and other clinicopathological features such as age and differentiation. Moreover, we found that the overall survival rate of patients with high UCA1 expression was significantly lower than that of patients with low UCA1 expression (*P* < 0.0001; Fig. 2). Together, these data indicate that UCA1 might promote the development of HSCC and be used as a prognostic marker for the disease.

**Table 1:** Clinicopathological characteristics of HSCC patients and relative expression of UCA1

Characteristics	Cases	UCA1 relative expression		*P* value
		Low	High	
Age, years				0.213
< 60	28	12	16	
≥ 60	25	15	10	
T category				0.034
T1-T2	22	15	7	
T3-T4	31	12	19	
Lymph node metastasis				0.020
N_0_	27	18	9	
N1-2	26	9	17	
Clinical stage				0.021
I-II	16	12	4	
III-IV	37	15	22	
Differentiation				0.613
Well	8	5	3	
Moderate	21	9	12	
Poor	24	13	11

### Influence of UCA1 depletion on proliferation, apoptosis, and cell cycle in FaDu cells

**Fig. 3:**
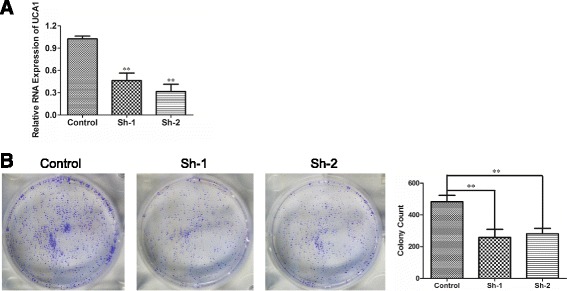
UCA1 depletion inhibited the proliferation of FaDu cells. **a** qRT-PCR was used to quantify the silencing efficiency of UCA1. *n* = 3; **P < 0.01. **b** Colony formation assays were used to assess the proliferation of FaDu cells transfected after culture for 14 days. Representative and quantitative results are shown. *n* = 3; **P < 0.01

Loss-of-function experiments in vitro were conducted to investigate the biological function of UCA1 in FaDu cells. The UCA1 expression levels of FaDu cells transfected with UAC1 siRNA (sh-1 or sh-2) were significantly lower than that of FaDu cells transfected with the scrambled sequence (Fig. 3a). The colony formation assay demonstrated that UCA1-depleted FaDu cells formed significantly fewer colonies than FaDu cells transfected with the scramble sequence did (Fig. 3b), indicating that UCA1 has a role in promoting the proliferation of FaDu cells.

**Fig. 4:**
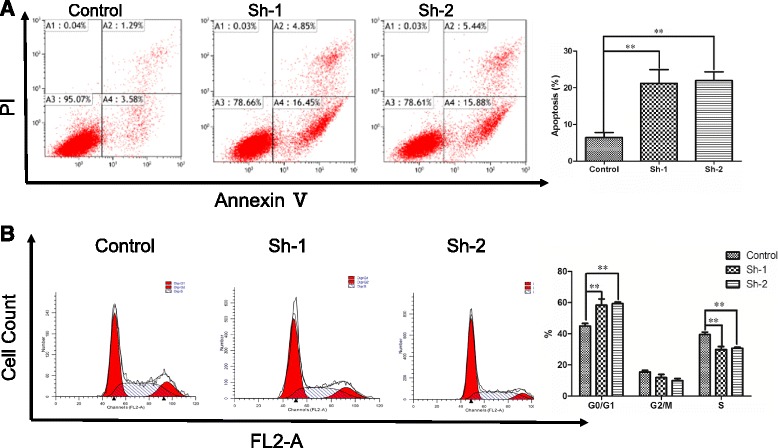
Silencing of UCA1 induced apoptosis and cell cycle arrest in FaDu cells. **a** Flow cytometry was used to assess the percentage of apoptotic FaDu cells after transfection for 5 days. Representative scatter plots and quantitative results are shown. *n* = 3; ** *P* < 0.01. **b** Flow cytometry was used to assess the cell cycle distribution of FaDu cells transfected for 24h and stained with PI. Representative and quantitative results are shown. *n* = 3; ** *P* < 0.01

We also studied whether UCA1 promotes the proliferation of FaDu cells by influencing apoptosis or cell cycle distribution. Flow cytometry revealed that lentiviral transfection with either UCA1 siRNA (sh-1 or sh-2) induced apoptosis in FaDu cells (Fig. 4a), indicating that UCA1 has an anti-apoptotic role. The cell cycle analysis revealed that, compared with transfection with the scrambled siRNA, transfection with either UCA1 siRNA (sh-1 or sh-2) caused a significantly larger accumulation of FaDu cells in G0/G1 phase and a reduction of the cells in S phase (Fig. 4b).

Given these findings, we hypothesized that the upregulation of UCA1 contributes to the proliferation of HSCC cells by exerting anti-apoptotic effects and promoting cell cycle progression.

### UCA1 contributes to metastasis by increasing the invasive ability of FaDu cells

**Fig. 5:**
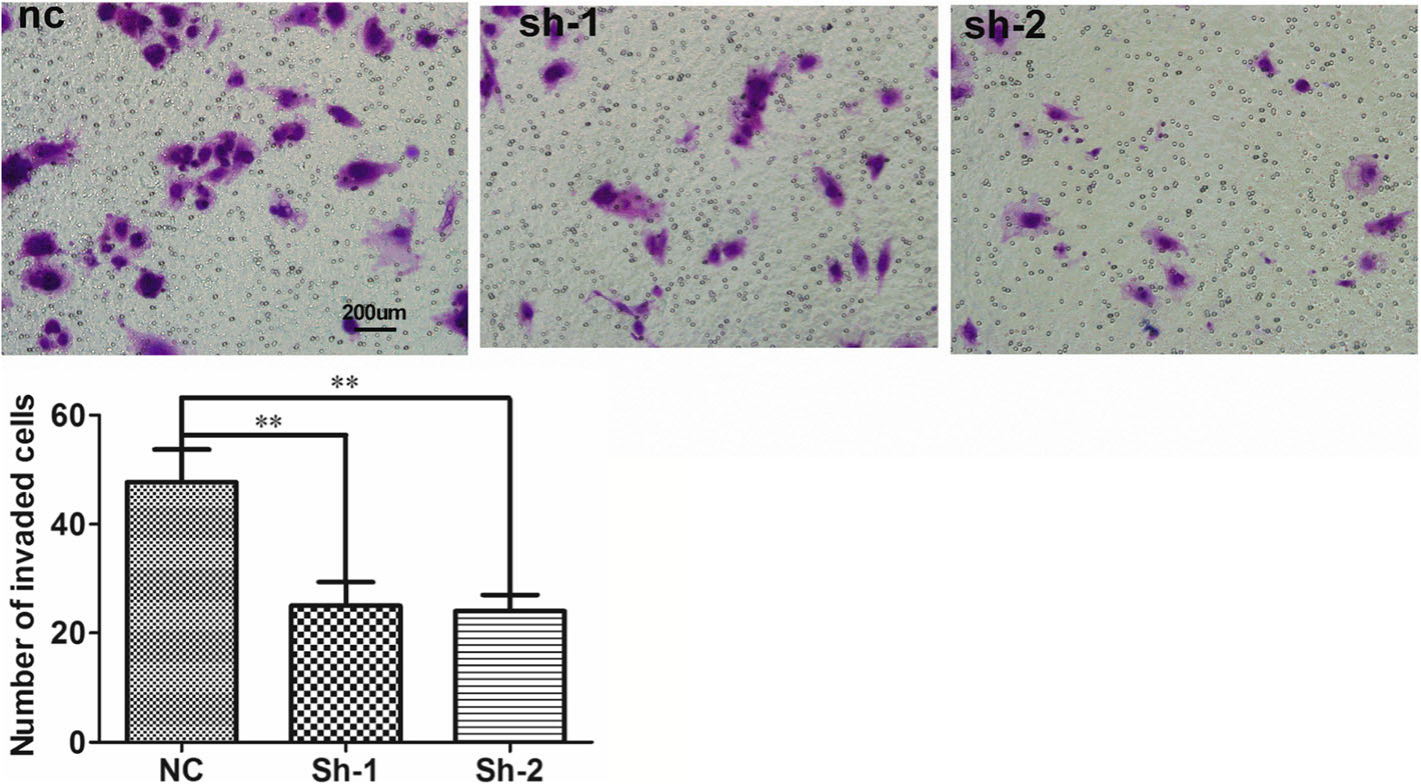
Knockdown of UCA1 weakened the invasive ability of FaDu cells. A transwell assay was conducted to assess the invasion of FaDu cells transfected with UCA1-siRNA (i.e. sh-1 and sh-2) and that of FaDu cells transfected with the scrambled control. Representative and quantitative results are shown. *n* = 3; ** *P* < 0.01

Considering that we found UCA1 expression to be significantly correlated with the lymph node metastasis of HSCC, we used transwell assays to further investigate the role of UCA1 in promoting HSCC invasion. The knockdown of UCA1 in FaDu cells significantly attenuated their invasive ability (Fig. 5), suggesting that UCA1 has a role in enhancing the invasion of HSCC cells. UCA1 was also identified as a pro-metastatic factor in HSCC cells.

## Discussion

HSCC, which is characterized by poor differentiation, early lymph node metastasis, and negligible symptoms in its early stages, is one of the worst head and neck squamous cell carcinomas. Despite advances in HSCC treatment strategies, HSCC patient outcomes remain quite poor [20]. The lack of early-stage diagnostic markers and novel therapeutic targets for HSCC emphasizes the need to quickly obtain an improved understanding the molecular mechanisms involved in HSCC development and progression.

With the development of sequencing technology, an increasing number of lncRNAs have been found to be differentially expressed in cancers and normal tissues. Among these differentially expressed lncRNAs, several are emerging as important regulators of essential biological processes in cancer, such as apoptosis, cell proliferation, cell cycle, and metastasis [21]. Among the transcripts our microarray data have revealed to be dysregulated [19], the lncRNA UCA1, which has an oncogenic role in several types of cancer, may help modulate the pathogenesis of HSCC [22].

Through validation experiments for microarray results, upregulation of UCA1 in HSCC tumor tissues was confirmed. Further, our analysis revealed that a high UCA1 expression level is significantly associated with advanced T category, late clinical stage, and worse prognosis, and these findings are consistent with those in other cancers, such as colorectal cancer and ovarian cancer. Further, our in vitro experiments revealed that UCA1 contributes to the proliferation of FaDu cells, likely by inhibiting apoptosis and facilitating cell cycle progression. These data partly explain the significance of UCA1 upregulation in HSCC tumor tissues and its relationship with related clinicopathological features. However, additional investigation is needed to identify the detailed molecular mechanisms underlying UCA1’s oncogenic roles.

Recently, several groups have presented evidence that UCA1 functions as a competing endogenous RNA in cancer cells. For example, Nie et al. reported that UCA1 was a tumor promoter in non–small cell lung cancer and that UCA1 upregulated the expression of the miR-193a-3p target gene ERBB4 through competitively “sponging” miR-193a-3p [23]. Wang et al. reported that the depletion of UCA1 inhibited the growth and metastasis of hepatocellular carcinoma cell lines in vitro and in vivo, probably by directly binding to miR-216b and downregulating miR-216b expression [24]. In addition, Brian et al demonstrated that UCA1 promoted the proliferation of colorectal cancer cells by physically binding to and inhibiting miR-204-5p [25]. These findings suggest that sponging certain miRNAs may be the underlying mechanism by which UCA1 is involved in the proliferation, apoptosis regulation, and the cell cycle of HSCC cells. In future studies, we will use TargetScan to search for potential miRNA targets of UCA1 and use RIP and qRT-PCR to identify them.

In accordance with others’ findings [22], we observed that UCA1 also promoted the metastasis of HSCC cells. Although UCA1 has been found to have a pro-metastatic role in several types of cancers, its pathway and downstream targets remain largely unknown. However, recent evidence has shown that UCA1 is involved with the Wnt6 and PI3 kinase/CREB pathways [26, 27], which are closely related to epithelial-to-mesenchymal transition. Thus, we thought that we could find the molecular mechanism of UCA1 regarding its pro-metastatic effect, with the increasingly enhanced understanding of its relevant pathways. **One of major limitations of this study is that our findings were observed for UCA1 in FaDu cells only, while it would be advantageous if the effect of more than one lncRNA is assessed in the both tumor samples and more than one cell line model. However, currently, in China, we only have one cell line of HSCC is available for in vitro study. Additionally, information on smoking and alcohol use should be included for adjustment for future prognosis analysis. Therefore, we will confirm our results in other HSCC cell lines and including smoking and alcohol information for adjustment in our future studies once they become available**.

Thus, the roles of UCA1 in HSCC warrant further study once other cell lines of HSCC become available.

## Conclusion

In conclusion, our study is the first to reveal the relationship between UCA1 expression and the clinical features of HSCC patients and demonstrate that UCA1 might serve as a prognostic biomarker of HSCC. We also identified an oncogenic role for UCA1 in the apoptosis, cell cycle, proliferation, and invasion in FaDu cells. These findings lay the foundation for future studies assessing the mechanisms of UCA1’s oncogenic actions.

## Abbreviations

lncRNA: Long noncoding RNA
HSCC: Hypopharyngeal squamous cell carcinoma
UCA1: Urothelial carcinoma-associated 1
qRT-PCR: Quantitative real-time PCR
MOI: Multiplicity of infection

## References

[1] Cooper JS, Porter K, Mallin K, Hoffman HT, Weber RS, Ang KK (2009). National Cancer Database report on cancer of the head and neck: 10-year update. Head Neck.

[2] Hall SF, Groome PA, Irish J, O’Sullivan B (2008). The natural history of patients with squamous cell carcinoma of the hypopharynx. Laryngoscope.

[3] Takes RP, Strojan P, Silver CE, Bradley PJ, Haigentz M, Wolf GT (2012). Current trends in initial management of hypopharyngeal cancer: the declining use of open surgery. Head Neck.

[4] Jang JY, Kim EH, Cho J, Jung JH, Oh D, Ahn YC (2016). Comparison of oncological and functional outcomes between initial surgical versus non-surgical treatments for hypopharyngeal cancer. Ann Surg Oncol.

[5] Guttman M, Rinn JL (2012). Modular regulatory principles of large non-coding RNAs. Nature.

[6] Jia H, Osak M, Bogu GK, Stanton LW, Johnson R, Lipovich L (2010). Genome-wide computational identification and manual annotation of human long noncoding RNA genes. RNA.

[7] Wilusz JE, Sunwoo H, Spector DL (2009). Long noncoding RNAs: functional surprises from the RNA world. Genes Dev.

[8] Arriaga-Canon C, Fonseca-Guzman Y, Valdes-Quezada C, Arzate-Mejia R, Guerrero G, Recillas-Targa F (2014). A long non-coding RNA promotes full activation of adult gene expression in the chicken alpha-globin domain. Epigenetics.

[9] Peter S, Borkowska E, Drayton RM, Rakhit CP, Noon A, Chen W (2014). Identification of differentially expressed long noncoding RNAs in bladder cancer. Clin Cancer Res.

[10] Prensner JR, Chinnaiyan AM (2011). The emergence of lncRNAs in cancer biology. Cancer Discov.

[11] Spizzo R, Almeida MI, Colombatti A, Calin GA (2012). Long non-coding RNAs and cancer: a new frontier of translational research?. Oncogene.

[12] Gibb EA, Brown CJ, Lam WL (2011). The functional role of long non-coding RNA in human carcinomas. Mol Cancer.

[13] Reis EM, Verjovski-Almeida S (2012). Perspectives of long non-coding RNAs in cancer diagnostics. Front Genet.

[14] Wang F, Li X, Xie X, Zhao L, Chen W (2008). UCA1, a non-protein-coding RNA up-regulated in bladder carcinoma and embryo, influencing cell growth and promoting invasion. FEBS Lett.

[15] Huang J, Zhou N, Watabe K, Lu Z, Wu F, Xu M (2014). Long non-coding RNA UCA1 promotes breast tumor growth by suppression of p27 (Kip1). Cell Death Dis.

[16] Han Y, Yang YN, Yuan HH, Zhang TT, Sui H, Wei XL (2014). UCA1, a long non-coding RNA up-regulated in colorectal cancer influences cell proliferation, apoptosis and cell cycle distribution. Pathology.

[17] Zheng Q, Wu F, Dai WY, Zheng DC, Ye H, Zhou B (2015). Aberrant expression of UCA1 in gastric cancer and its clinical significance. Clin Transl Oncol.

[18] Li JY, Ma X, Zhang CB (2014). Overexpression of long non-coding RNA UCA1 predicts a poor prognosis in patients with esophageal squamous cell carcinoma. Int J Clin Exp Pathol.

[19] Zhou J, Li W, Jin T, Xiang X, Li M, Wang J (2015). Gene microarray analysis of lncRNA and mRNA expression profiles in patients with hypopharyngeal squamous cell carcinoma. Int J Clin Exp Med.

[20] Zhou J, Li M, Yu W, Li W, Wang J, Xiang X (2016). AB209630, a long non-coding RNA decreased expression in hypopharyngeal squamous cell carcinoma, influences proliferation, invasion, metastasis, and survival. Oncotarget.

[21] Huarte M (2015). The emerging role of lncRNAs in cancer. Nat Med.

[22] He A, Hu R, Chen Z, Liao X, Li J, Wang D (2016). Role of long noncoding RNA UCA1 as a common molecular marker for lymph node metastasis and prognosis in various cancers: a meta-analysis. Oncotarget.

[23] Nie W, Ge HJ, Yang XQ, Sun X, Huang H, Tao X (2016). LncRNA-UCA1 exerts oncogenic functions in non-small cell lung cancer by targeting miR-193a-3p. Cancer Lett.

[24] Wang CC, Liu HE, Lee YL, Huang YW, Chen YJ, Liou JP (2016). MPT0B169, a novel tubulin inhibitor, induces apoptosis in taxol-resistant acute myeloid leukemia cells through mitochondrial dysfunction and Mcl-1 downregulation. Tumour Biol.

[25] Bian Z, Jin L, Zhang J, Yin Y, Quan C, Hu Y (2016). LncRNA-UCA1 enhances cell proliferation and 5-fluorouracil resistance in colorectal cancer by inhibiting miR-204-5p. Sci Rep.

[26] Fan Y, Shen B, Tan M, Mu X, Qin Y, Zhang F (2014). Long non-coding RNA UCA1 increases chemoresistance of bladder cancer cells by regulating Wnt signaling. FEBS J.

[27] Wang Y, Chen W, Yang C, Wu W, Wu S, Qin X (2012). Long non-coding RNA UCA1a (CUDR) promotes proliferation and tumorigenesis of bladder cancer. Int J Oncol.

